# Questioning Aerosol Transmission of Influenza 

**DOI:** 10.3201/eid1301.061202

**Published:** 2007-01

**Authors:** Camille Lemieux, Gabrielle Brankston, Leah Gitterman, Zahir Hirji, Michael Gardam

**Affiliations:** * University Health Network, Toronto, Ontario, Canada; † University of Toronto, Toronto, Ontario, Canada; 1 Currently with the Durham Region Health Department, Whitby, Ontario, Canada

**Keywords:** influenza, transmission, droplet, aerosol, letter

## To the Editor 

We have reviewed the literature cited in Tellier’s Review of Aerosol Transmission of Influenza A Virus (*1*) and disagree that it supports the conclusions drawn regarding the importance of aerosols in natural influenza infection. In certain cited studies, researchers recovered viable virus from artificially generated aerosols; this is not evidence that aerosol transmission leads to natural human infection (*2*,*3*). By standard definitions, the rarity of long-range infections supports the conclusion that effective aerosol transmission is absent in the natural state (*4*) (www.cdc.gov/ncidod/dhqp/gl_isolation_hicpac.html). The superior efficacy of inhaled versus intranasal zanamivir is referenced as support for the idea that the lower respiratory tract is the preferred site of influenza infection; however, 1 study cited is insufficiently powered, and the other 2 do not compare the intranasal and inhaled routes (*5*–*7*). The major site of deposition of inhaled zanamivir is the oropharynx (77.6%), not the lungs (13.2%) (www.gsk.ca/en/products/prescription/relenza_pm.pdf). In another flawed study (*8*), study participants naturally infected with wild-type virus are compared with study participants experimentally infected with an attenuated strain.

In a review of such relevance, critical analysis of confounding factors is necessary. The Alaska Airlines outbreak (*9*) is presented as proof of airborne influenza transmission; however, droplet/contact transmission remains plausible because passenger movement was not restricted and the index patient was seated in high-traffic area. In the Livermore Hospital study (*10*), serious confounders such as bed arrangements, number of influenza exposures, patient mix, and ventilation were not accounted for.

We encourage readers of Teller’s article to review the relevant primary literature. We believe that the only reasonable conclusion that can be drawn at this time is that aerosol transmission does not play a major role in natural influenza epidemiology. Whether aerosols play any role in the transmission of influenza is a question demanding an answer; it is clear that we do not yet have that answer.
