# Supplementary material for: Questioning Aerosol Transmission of Influenza
Source: Emerg Infect Dis. 2007 Jan;13(1):173–5. doi: 10.3201/eid1301.061202 (PMC2725811; doi:10.3201/eid1301.061202)
Supplement: Related texts

# Reply: In Response

**Authors:** Raymond Tellier

**Affiliations:** * Hospital for Sick Children, University of Toronto, Toronto, Canada

Coughing and sneezing during influenza produce virus-containing aerosols. In the laboratory, influenza virus in homogeneous aerosols, free of large droplets, can infect volunteers at very small doses; studies of infectivity decay in aerosols show persistence for hours. These observations required the generation of artificial aerosols but were performed under conditions that do not enhance stability or virulence (*1**,**2*). Therefore, they have great relevance for natural infections.

The scarcity of infections that are transmitted long range in well-ventilated areas does not rule out infectivity of aerosol-size particles near patients. That only 13% of inhaled zanamivir is deposited in the lungs is not important: after inhalation, the zanamivir concentration throughout the respiratory tract is >10 μmol/L, orders of magnitude above the 50% inhibitory concentration (*3*). Intranasal zanamivir is protective against large droplets (*4*), which are trapped in the nose (*5*). The requirement for inhaled zanamivir in natural infections (*6**,**7*) points to aerosol contribution and to the lower respiratory tract as the preferred site.

Little et al. (*8*) compared the severity of natural illness caused by H3N2 strains from 1974 and 1975 to that caused by experimental intranasal inoculation from H3N2 strains from 1972, 1974, and 1975. The challenge strains underwent few passages; characterizing them as "attenuated" is incorrect.

Although large droplets probably accounted for some cases in the Alaska Airlines outbreak (*9*), this outbreak was remarkable for its high attack rate (72%) and for deficient ventilation, which would increase transmission by aerosols but not by large droplets. Passengers with influenza are common, yet with proper ventilation such an attack rate is uncommon.

During the Livermore Hospital study (*10*), respiratory infections other than influenza occurred in both groups. It was assumed that visitors and staff would provide equivalent introductions of the virus during the several months of the study; 4 study participants in the irradiated building seroconverted, but the virus did not propagate. The concern by Lemieux and colleagues about ventilation is odd because it would affect mostly aerosol transmission.

I concur with encouraging readers to review the original references. They make a compelling case for the importance of aerosol transmission. In contrast, no convincing data rule it out.

## References

[1] Douglas RG. Influenza in man. In: Kilbourne ED, editor. The influenza viruses and influenza. New York: Academic Press; 1975. p. 375–447.

[2] Schaffer FL, Soergel ME, Straube DC. Survival of airborne influenza virus: effects of propagating host, relative humidity, and composition of spray fluids. Arch Virol. 1976;51:263–73. 10.1007/BF01317930987765

[3] Cass LM, Brown J, Pickford M, Fayinka S, Newman SP, Johansson CJ, Pharmacoscintigraphic evaluation of lung deposition of inhaled zanamivir in healthy volunteers. Clin Pharmacokinet. 1999;36(Suppl 1):21–31. 10.2165/00003088-199936001-0000310429837

[4] Calfee DP, Peng AW, Hussey EK, Lobo M, Hayden FG. Safety and efficacy of once daily intranasal zanamivir in preventing experimental human influenza A infection. Antivir Ther. 1999;4:143–9.12731753

[5] Knight V. Airborne transmission and pulmonary deposition of respiratory viruses. In: Hers JF, Winkles KC, editors. Airborne transmission and airborne infections VIth International Symposium on Aerobiology. New York: Wiley; 1973. p. 175–82.

[6] Kaiser L, Henry D, Flack NP, Keene O, Hayden FG. Short-term treatment with zanamivir to prevent influenza: results of a placebo-controlled study. Clin Infect Dis. 2000;30:587–9. 10.1086/31369610722450

[7] Hayden FG, Gubareva LV, Monto AS, Klein TC, Elliot MJ, Hammond JM, Inhaled zanamivir for the prevention of influenza in families. Zanamivir Family Study Group. N Engl J Med. 2000;343:1282–9. 10.1056/NEJM20001102343180111058672

[8] Little JW, Douglas RG Jr, Hall WJ, Roth FK. Attenuated influenza produced by experimental intranasal inoculation. J Med Virol. 1979;3:177–88. 10.1002/jmv.1890030303479857

[9] Moser MR, Bender TR, Margolis HS, Noble GR, Kendal AP, Ritter DG. An outbreak of influenza aboard a commercial airliner. Am J Epidemiol. 1979;110:1–6.46385810.1093/oxfordjournals.aje.a112781

[10] McLean RL. Discussion after paper: the mechanism of spread of Asian influenza. Am Rev Respir Dis. 1961;83:36–8.10.1164/arrd.1961.83.2P2.2913790691
